# Consumption Patterns and the Nutritional Contribution of Total, Processed, Fresh, and Fresh-Lean Pork to the U.S. Diet

**DOI:** 10.3390/nu15112595

**Published:** 2023-06-01

**Authors:** Lindsay Y. Datlow, Mark Leventhal, Jay King, Taylor C. Wallace

**Affiliations:** 1 SAS Institute, Cary, NC 27513, USA; 2 Think Healthy Group, LLC, Washington, DC 20001, USA; 3 Department of Nutrition and Food Studies, George Mason University, Fairfax, VA 22030, USA; 4 Friedman School of Nutrition Science and Policy, Tufts University, Boston, MA 02111, USA

**Keywords:** pork meat, red meat, diet, nutritional status, food, processed, nutrition surveys

## Abstract

Pork has the potential to provide several macro and micronutrients to the diet, as it is a commonly consumed protein in the United States and across many cultures worldwide. There is an absence of clinical and observational studies that isolate the nutritional contribution of various types of pork intake from that of other red and/or processed meats. The objective of this study was to assess consumption patterns and the nutritional contribution of total, processed, fresh, and fresh-lean pork to the diets of participants aged 2+ years enrolled in the National Health and Nutrition Examination Survey (NHANES) 2007–2018 data cycles. The recent National Cancer Institute method was used to disaggregate fresh and processed pork intake from the USDA Food Patterns Equivalents Database. The mean intake of total pork among consumers was estimated to be 79.5 ± 0.82, 54.2 ± 0.69, 54.6 ± 0.93, and 45.9 ± 0.73, g/d for men, women, boys, and girls, respectively. Total pork consumption subtly increased intakes of total energy and several macro and micronutrients, decreased diet quality (HEI-2015) scores (adults only), and consumption of other “healthful” food groups. Only subtle but clinically insignificant effects of pork intake on biomarkers of nutritional status were shown. These trends were largely driven by processed pork consumption and the co-consumption of foods such as condiments. Increasing the availability and education around fresh-lean cuts may help to increase intake of protein and other key nutrients across certain subpopulations, without adversely affecting diet quality and biomarkers of health status.

## 1. Introduction

Food-based dietary guidelines in the U.S. and around the globe encourage consumption of a variety food groups to meet nutrient needs [1,2]. Pork ranks first in global per capita meat consumption according to the U.S. Department of Agriculture (USDA) Foreign Agriculture Service [3], but is the third most commonly consumed meat in the United States, following chicken and beef, according to the USDA Economic Research Service’s Food Availability (per capita) Data System [4]. Changes in the pork industry over the past few decades have increased availability of fresh-lean cuts available to consumers; seven cuts now meet USDA standards to be labeled as “lean” cuts [5]. Additionally, both pork tenderloin and sirloin meet the American Heart Association’s Heart Health Checkmark criteria, which means they contain ≤5 g fat, ≤2 g saturated fat, and ≤480 mg sodium per serving. Leaner cuts of pork have been shown to be similar in their nutritional composition to that of skinless turkey or chicken breast [6]. After obtaining gastric and jejunal contents obtained from rats fed four meat proteins (pork, beef, chicken, and fish), the pork and beef samples had a greater number of fragments in vivo, compared to chicken and fish. Further, using an additional in vitro model, the authors describe the digestibility of pork proteins being higher for pork proteins and lowest for beef proteins [7]. The USDA FoodData Central database lists a 4-ounce raw, boneless, lean pork chop (top-loin) (168251) as providing 144 kcal, 25.3 g protein, 3.86 g fat (1.36 g saturated fat), and substantial amounts of iron, zinc, selenium, magnesium, phosphorus, potassium, thiamin, riboflavin, niacin, pantothenic acid, choline, vitamin B_6_, and vitamin B_12_ to the diet [8]. 

From a sustainability standpoint, diets that replace ruminants with other alternatives such as fish, poultry, and pork have been shown to have a reduced environmental impact, but to a lesser extent than plant-based alternatives [9]. However, a strictly plant-based diet does not provide adequate amounts of certain limiting amino acids and several micronutrients predominantly derived and bioavailable from animal-sourced foods. For example, data consistently show substitution of animal-source proteins with plant-source proteins has detrimental impacts on bone health and risk of fractures [10,11,12]. A serving of pork emits less than 10% of the greenhouse gas emissions of beef and is more akin to that of poultry, fish, eggs, and dairy [13]. 

Our research group recently published a scoping review that found an dearth of clinical and observational studies that isolate the nutritional contribution of various types of pork intake from that of other red and/or processed meats [14]. In particular, the scientific literature remains unclear on the role that fresh and fresh-lean pork products may have on the nutritional status of individuals. A recent modeling analysis of the U.S. Thrifty Food Plan showed fresh pork to be the preferred meat source, providing high-quality protein at the lowest cost [15]. It is possible that certain types of pork may act as “carrier foods” [16] that increase intake of other food groups in the diet. Therefore, the objective of this research was to assess consumption patterns and nutritional contribution of total, processed, fresh, and fresh-lean pork to the diets of participants enrolled in the U.S. National Health and Nutrition Examination Survey (NHANES), 2007–2018 data cycles.

## 2. Materials and Methods

### 2.1. Study Design and Population

The National Center for Health Statistics (NCHS) of the U.S. Centers for Disease Control and Prevention (CDC) administers the NHANES, a nationally representative, continuous, cross-sectional survey of community-dwelling civilian U.S. residents, on an annual basis [17]. The NHANES survey protocol was approved by the Research Ethics Review Board of the NCHS and written informed consent was obtained for all survey participants and proxies. Data from the NHANES 2007–2018 were combined in these analyses, unless otherwise noted below. Participants 2+ years of age with complete data from the two 24-h dietary recall interviews were included in these analyses. Subjects who were pregnant or lactating and those with extreme caloric intakes <500 and >5000 kcal per day were excluded from these analyses. Since 1999, the NHANES protocol has included an in-person household interview component and follow-up health examination via the mobile examination center (MEC) for each participant. Proxy respondents provided information for young children and proxy-assisted interviews were utilized for children aged 6–11 years. Further details of the NHANES can be found on the NCHS website [17].

### 2.2. Demographic Characteristics

All demographic data used for these analyses, including data on age, sex, race, and ethnicity, household income, and family size were obtained from participants or proxies using the computer-assisted personal interview system during the household interview. Age was categorized to correspond with the Dietary Reference Intake (DRI) age groups, defined as 2–3, 4–8, 9–13, 14–18, 19–30, 31–50, 51–70, and 71+ years. Children and adults were defined as those individuals aged 2–18 years and ≥19 years, respectively. Gender was defined as male or female. Race and ethnicity were defined as Hispanic, non-Hispanic white, non-Hispanic black, non-Hispanic Asian, and other (includes mixed races). The percent poverty-income ratio (%PIR) was calculated using the household income and family size variables and categorized as low (<130%), medium (131–300%), and high (≥300%), as previously described [18]. Food security status was categorized as high, marginal, low, and very low. 

### 2.3. Dietary Intake

What We Eat In America (WWEIA) is the dietary intake interview component of NHANES that is conducted as a partnership between the USDA and NCHS. Two days of 24-h dietary recall interview data are collected through an initial in-person interview in the MEC and the second interview over the phone within 3–10 days, as previously described [19]. WWEIA data are collected by trained interviewers using the UDSA Automated Multiple-Pass Method [20]. The Automated Multiple-Pass Method is a fully computerized and validated method for collecting 24-h dietary recalls either in-person or by telephone [21,22]. Each participant reported the source of each food item (i.e., where purchased or otherwise obtained) and we grouped these settings for this analysis as (1) grocery stores, supermarkets, or other stores; (2) restaurants; (3) schools; and (4) other (e.g., entertainment facility, sports or recreation facility, etc.), as previously published by Liu et al. 2021 [23]. The USDA Food Patterns Equivalents Database was used to convert the foods and beverages into 37 USDA Food Patterns components, as previously described [16,24]. 

### 2.4. Disaggregation of Processed Pork and Definitions of Lean and Fresh

Processed pork was disaggregated from the USDA Food Patterns Equivalents Database based using the definition and method by O’Connor et al. (2022), as well as the SAS program provided by the National Cancer Institute (NCI) [25]. Lean was defined as pork containing less than 10 g fat, 4.5 g saturated fat, and 95 mg cholesterol per serving, per USDA. Fresh pork was defined as muscle per the American Meat Science Association Lexicon [26]. We did not include intake of pork organ meat in this paper, as sources (e.g., liver) were not uniformly categorized being derived from pork vs. another source, reported consumption was extremely negligible, and our overall focus was on muscle cuts.

### 2.5. Estimation of Usual Nutrient Intakes

The USDA Food and Nutrient Database for Dietary Studies was used to convert foods and beverages (as reported) to their respective macro and micronutrient intake values [27]. The National Cancer Institute method, as previously described, was used to estimate usual macro and micronutrient intakes from the diet. SAS macros used to fit this model and to perform the estimation of intake distributions, as well as additional details regarding the NCI method are available on the NCI website [28]. The fitted model is a 2-part model that uses logistic regression for estimating probability of intake for each participant, and then a linear regression to estimate the amount of intake per day, while accounting for between-person variation [28].

The Dietary Reference Intakes (DRIs) are a family of nutrient values defined by the National Academy of Medicine, intended to serve as a guide for good nutrition and provide the basis for development of dietary guidelines in the U.S. and Canada. The prevalence of inadequate dietary intakes was determined using the EAR cut-point method, as previously described and was reported as the percentage with usual intakes below the Estimated Average Requirement (EAR) [29]. The percentage of the population with usual intakes above the tolerable upper intake level (UL) was also determined in our analyses. Mean dietary intakes of macro and micronutrients and the proportion of the population that met the EAR and exceeded the UL were compared by computing a z-statistic.

### 2.6. Diet Quality

Diet quality scores were determined using the USDA Healthy Eating Index-2015 (HEI-2015), which assesses the alignment of dietary intake with recommendations of the 2015–2020 Dietary Guidelines for Americans [30]. The HEI-2015 comprises of 13 component scores. Total component scores range between 0 and 100, with a higher score reflecting greater alignment to the recommendations of the 2015–2020 Dietary Guidelines for Americans [30,31]. Covariates used in the analyses included sex, age, and PIR. The scores were calculated at group level using the multivariate Markov Chain Monte Carlo (MCMC) approach, based on two-days of dietary recalls using the publicly available SAS macros from the NCI [32].

### 2.7. Biomarkers of Nutritional Status

NHANES participants who provided biospecimen collection (e.g., blood, urine, and other biological samples for laboratory analyses) and for which nutrient biomarker data were available were utilized in these analyses. The data cycles from select NHANES data cycles were used to assess nutritional biomarkers (after exclusions), as this data was not collected in all releases (Table 1). 

Natural logarithm transformations were used due to the right-skewedness of the nutritional biomarkers. Geometric means ± SE are presented for nutritional biomarkers. Linear regression analyses on log-transformed biomarkers were performed to compare nutritional biomarkers among non-consumers and consumers of total pork, fresh pork, fresh-lean pork, and processed pork.

### 2.8. Statistical Analyses

All statistical analyses were performed using SAS software (version 9.4; SAS Institute, Inc.; Cary, NC, USA). Significance was set at *p* ≤ 0.05. All statistical analyses were adjusted for survey design and weighting variables to account for the complex sampling design of NHANES and to ensure nationally representative estimates. Descriptive statistics and measures of variability were generated for all variables. A Student’s *t*-test or Wilcoxin rank-sum test was used to compare the mean levels of continuous variables. A Pearson chi-squared test or Fisher’s exact test was performed to compare the distribution of the categorical variables.

## 3. Results

**Figure 1:**
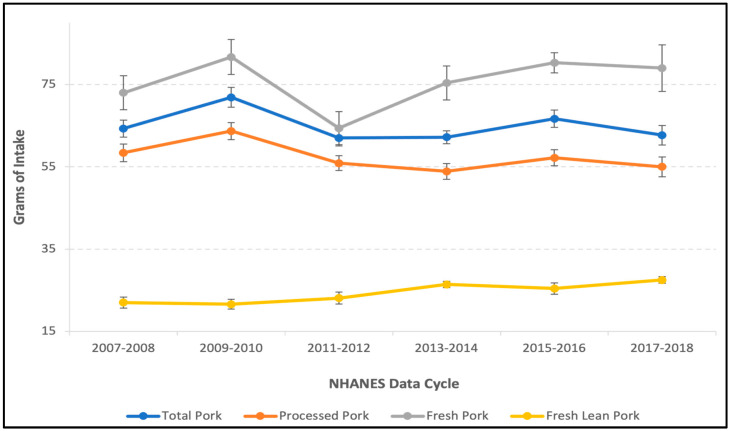
Trends in pork intake over 10-years (adults and children combined).

The mean quantity of total pork intake among consumers was estimated to be 79.5 ± 0.82, 54.2 ± 0.69, 54.6 ± 0.93, and 45.9 ± 0.73, g/d for men, women, boys, and girls, respectively (Table 1). Men and boys had significantly higher intakes of total, processed, fresh, and fresh-lean pork, compared to women and girls of the same age group. Processed pork was the most frequently consumed type of pork. In those who reported pork intake, about 87.4% and 92.4% of adults and children reported consuming processed pork. In those who reported pork intake, about 21.8% and 14.6% of adults and children reported consuming fresh pork, whereas about 9.60% and 8.30% of adults and children reported consuming fresh-lean pork. The quantity of total pork consumed increased with age among consumers, plateauing at age 31–50 years in both men (86.8 ± 1.76 g/d) and women (55.8 ± 1.02 g/d), and then decreasing with age in older adulthood. The quantity of processed and fresh pork consumed followed similar patterns with age as total pork; however, the quantity of fresh-lean pork consumed was similar across age groups (Supplemental Table S1). The quantity of total pork consumed among consumers (adults and children combined) stayed relatively stable between the 2007–2008 and 2017–2018 NHANES data cycles, whereas the quantity of fresh-lean pork increased among consumers (Figure 1).

Quantities of total, processed, fresh, and fresh-lean pork consumed among adult consumers did not differ by PIR. Quantities of total (56.6 ± 2.02 vs. 49.7 ± 1.41 g/d), processed (51.9 ± 2.09 vs. 44.3 ± 1.21 g/d), and fresh (68.7 ± 5.69 vs. 58.3 ± 2.95 g/d) pork consumed was higher among child consumers with >300 PIR, compared to those with <130 PIR, respectively. There was no significant difference in the quantity of fresh-lean pork consumed among child consumers based on PIR (Supplemental Table S2). The quantities of total, processed, fresh, and fresh-lean pork consumed by adult consumers did not differ based upon food security status in adults. The quantity of processed pork consumed by child consumers with low and very-low food security status was lower than those with high food security status (40.8 ± 1.49 vs. 44.1 ± 2.55 vs. 49.7 ± 1.64 g/d), respectfully. Quantities of total, fresh, and fresh-lean pork consumed did not differ among child consumers based on food security status (Supplemental Table S3).

The quantities of total pork consumed by Hispanic consumers was lower (55.2 ± 0.90 g/d) compared to other racial and ethnic groups. Hispanics and non-Hispanic Asian consumers had the lowest intakes of processed pork, 45.4 ± 0.78 and 48.0 ± 1.89 g/d, respectfully. Non-Hispanic Black and non-Hispanic Asian consumers had the lowest intakes of fresh pork, 64.5 ± 1.99 and 65.7 ± 3.08 g/d, respectively. Non-Hispanic Asian consumers had higher intakes of fresh-lean pork (27.5 ± 1.06 g/d) (Supplemental Table S4).

Among adult consumers of total, processed, and fresh pork, the greatest quantities of intake were derived from grocery stores, followed by other sources and restaurants (*p* < 0.01). Grocery stores and other sources remained the top sources of total and processed pork consumption in children (*p* < 0.01). In children, consumption of fresh-lean pork was not significantly different by source (*p* > 0.05) (Table 2).

Girls (2–18 years) who consumed pork had subtle but non-clinically relevant increased intakes of total calories, carbohydrates, protein, fat, saturated fat, MUFA, PUFA, cholesterol, vitamin C, vitamin D, thiamin, riboflavin, niacin, vitamin B12, choline, calcium, potassium, sodium, phosphorus, iron, zinc, and selenium, compared to non-consumers. There were not any clinically relevant differences in estimated nutrient intakes among girtls who were consumers of processed, fresh, or fresh-lean pork compared to total pork consumers (Supplemental Table S5). Boys (2–18 years) who consumed pork had subtle but non-clinically relevant increased intakes of total calories, carbohydrates, protein, fat, saturated fat, MUFA, PUFA, cholesterol, vitamin K, thiamin, riboflavin, vitamin B12, choline, calcium, potassium, sodium, phosphorus, zinc, copper, and selenium, compared to non-consumers. There were not any clinically relevant differences in estimated nutrient intakes among boys who were consumers of processed, fresh, or fresh-lean pork compared to total pork consumers, except for sodium. Intakes of sodium were lower among boys who consumed fresh-lean pork (3361 ± 14.6 mg/d) compared to those who consumed total (3507 ± 14.6 mg/d), processed (3517 ± 15.1 mg/d), or fresh pork (3526 ± 34.3mg/d) (Supplemental Table S6). Adult women who consumed pork had subtle but non-clinically relevant increased intakes of total calories, carbohydrates, protein, fat, saturated fat, MUFA, PUFA, cholesterol, thiamin, riboflavin, niacin, vitamin B12, choline, calcium, sodium, phosphorus, iron, zinc, and selenium, and decreased intaks of fiber, vitamin A, vitamin C, vitamin K, and magnesium, compared to non-consumers. There were not any clinically relevant differences in estimated nutrient intakes among adult women who were consumers of processed, fresh, or fresh-lean pork compared to total pork consumers (Supplemental Table S7). Adult men who consumed pork had subtle but non-clinically relevant increased intakes of total calories, carbohydrates, protein, fat, saturated fat, MUFA, PUFA, cholesterol, vitamin A, vitamin D, thiamin, riboflavin, niacin, vitamin B6, folate, vitamin B12, choline, calcium, potassium, sodium, phosphorus, iron, zinc, copper and selenium, and decreased intakes of vitamin C, compared to non-consumers. There were not any clinically relevant differences in estimated nutrient intakes among adult men who were consumers of processed, fresh, or fresh-lean pork compared to total pork consumers (Supplemental Table S8). Pork intake was generally associated with a subtle decreased percent of individuals who did not meet the Estimated Average Requirement (EAR) for several nutrients and did not significantly contribute to the percent of individuals who exceeded the UL (Supplemental Tables S5–S8). Consumers of pork had slightly lower intakes of fruits, vegetables, dark green vegetables, and whole grains, and slightly higher intakes of refined grains, total protein foods, meat, total dairy, and added sugars. Processed and fresh-lean pork consumers followed similar trends but had a slightly lower intake of meat, compared to non-consumers. Fresh pork consumers also followed similar trends but had a slightly higher intake of vegetables. There was not any significant difference in the quantity of vegetables and total dairy between consumers of fresh-lean pork and non-consumers (Supplemental Table S9). The 10 top foods co-consumed alongside pork include (1) lettuce, (2) tomatoes, (3) ketchup, (4) mayonnaise, (5) mustard, (6) white bread, (7) bananas, (8) apples, (9) rolls (white), and (10) American cheese. Processed pork consumers followed similar trends. Fresh and fresh-lean pork consumers more frequently co-consumed bananas, salsa (red), apples, and white rice, whereas condiments fell further down the list. Lettuce and tomatoes were still the most commonly co-consumed foods alongside fresh and fresh-lean pork (Supplemental Table S10). 

Pork consumers of all ages had slightly lower diet quality (HEI-2015) scores compared to non-consumers, except 2–3-year-old girls, in which there was no significant difference. Processed pork followed similar trends. Decreases in diet quality scores were more subtle, and in several gender/age groups not statistically significant, among fresh and fresh-lean pork consumers compared to non-consumers (Table 3). 

Pork consumers had slightly lower but non-clinically relevant circulating levels of vitamin B_12_ (379 ± 2.76 vs. 398 ± 4.84 pmol/L), vitamin D (64.1 ± 0.65 vs. 65.1 ± 0.61 nmol/L), vitamin E (1108 ± 13.7 vs. 1144 ± 8.51 µmol/L), and copper (113 ± 0.99 vs. 116 ± 1.05 µmol/L), and slightly higher but non-clinically relevant levels of iron (79.8 ± 0.42 vs. 78.7 ± 0.46 µg/dL), compared to non-consumers. Processed pork consumers exhibited similar trends but had slightly higher but non-clinically relevant urinary iodine levels (144 ± 2.71 vs. 138 ± 2.51 µg/L). Nutritional biomarkers were not different among consumers of fresh and fresh-lean pork compared to non-consumers, except for slightly but non-clinically relevant lower vitamin D (fresh and fresh-lean pork), RBC folate (fresh-lean pork only) and vitamin E levels (fresh-lean pork only) (Supplemental Table S4).

## 4. Discussion

We identified consumption patterns and the nutritional contribution of total, processed, fresh, and fresh-lean pork to the diets of Americans; however, intake did not seem to greatly (or consistently) affect usual nutrient intakes, diet quality, and biomarkers of nutritional status in this nationally representative cross-sectional analysis of the U.S. population. Our intake data are consistent with that from Murphy et al. 2011 [33] and An et al. 2020 [34] which show that fresh pork and fresh lean pork account for only a small portion of the total pork consumed by Americans. Relative to beef, the amount and proportion of consumers who self-report intake of fresh and fresh-lean pork is lower and less frequent than beef [35,36]. This may be due to the increased availability of fresh and fresh-lean beef options currently on the market. Similar age and sex intake patterns among pork consumers have been reported for total animal protein, beef, and red meat intakes [35,36,37,38]. Our data demonstrate that fresh lean pork intakes have gradually increased, whereas processed pork intake seems to have gradually decreased over the past decade. This may be due in part to a growing amount of public health campaigns that encourage consumers to moderate intake of over-consumed nutrients such as sodium and saturated fat, as well as the increasingly negative consumer perception of processed foods and, in particular, processed red meat products. Data from the International Food Information Council (IFIC) show that approximately 44% of consumers perceive processed foods as negative due to their “impact on health” [39]. Additionally, these data also show that about one-third of Americans self-report either decreasing intake or not consuming red meat in the last year [39].

Among U.S. children who consumed pork, those in the lowest versus the highest PIR group had decreased intakes, with the exception of fresh-lean pork. A similar pattern was shown in children with very low versus high food security status. A recent modeling study of the U.S. Thrifty Food Plan suggests pork to be the preferred meat source, providing high-quality protein at the lowest cost [15]. Thus, one could hypothesize that the nutritional benefits of fresh-lean pork might be greater among children who are socioeconomically disadvantaged and/or more food insecure. Cultural differences in pork intake patterns also exist, as evidenced by differences among racial and ethnic groups. Consistent with our current analyses of the Korean National Health and Nutrition Examination Survey (KNHANES) [40], the non-Hispanic Asian population in the U.S. who consumed pork self-reported higher intakes of fresh-lean pork and lower intakes of processed pork.

The 2020–2025 DGA has evolved towards focusing on healthy dietary patterns as a whole rather than on individual nutrients, foods, or food groups in isolation [1]. Previous iterations of the DGA highlighted under-consumed, also known as “shortfall nutrients”, or represent those that are consumed by individuals in amounts below the EAR or Adequate Intake (AI) [31]. The shortfall nutrients identified by the 2015–2020 DGA include potassium, dietary fiber, choline, magnesium, calcium, and vitamins A, D, E, and C. Iron was also considered a shortfall nutrient among premenopausal women, 19–50 years of age [31]. While these 10 nutrients are known to be under-consumed across the board, only four—calcium, potassium, vitamin D, and dietary fiber—are considered in current and former iterations of the DGA as “nutrients of public health concern”, since their low intakes are linked to critical health problems, for example, the relationship of high blood pressure with low potassium intake [41]. Iron is considered a nutrient of public health concern for women who are pregnant. [1,31] Pork intake was associated with increases in several nutrients in the American diet, including many that are considered shortfall nutrients and/or nutrients of public health concern [41]. A similar analysis of NHANES, found lean beef to contribute substantial amounts of total energy and nutrients such as protein, fat, saturated fat, MUFA, PUFA, cholesterol, riboflavin, niacin, vitamin B6, vitamin B12, iron, zinc, phosphorus, sodium, potassium, and magnesium to the diet [42]. These data reflect some similarities in nutrients derived from animal-source foods, but also the higher consumption rates and quantities of beef compared to pork across the U.S. population. Approximately 50% and 41% of adults, aged 19–50 and 51+ years have been shown to consume lean beef on a given day [42].

Pork intake resulted in overall increases in energy intake, subtle increases in several nutrient intakes, and subtle decreases in diet quality (HEI-2015) scores, particularly among adults; biomarkers of nutritional status remained similar in consumers vs. non-consumers. The decrease in diet quality among adults is likely impacted by other foods consumed alongside pork within the typical American diet. For example, condiments and white bread were shown to be popular co-consumed foods alongside pork, and therefore, increases in overconsumed nutrients and food groups like sodium and refined grains may be associated with intake. Our group recently defined a “carrier food” as those foods that may indirectly influence nutrient intakes and diet quality [16]. In contrast, pork intake is associated with increased vegetable intake with no influence on diet quality scores among Korean participants enrolled in the KNHANES [40]. The differences in consumption patterns between Americans and Koreans reflect the need for increased consumer advocacy and education in the U.S. on how to better utilize pork as part of a healthful and balanced diet.

Our study had many strengths. NHANES is a large nationally representative database. The utilization the SAS program created by O’Connor et al. (2022) [25] increases replicability and comparability of our findings in future investigations by standardizing exposure assessments. This study was not without limitations. Analyses of NHANES are limited by the cross-sectional design that precludes cause-effect interferences. NHANES also relies on self-reported dietary intake, and therefore the data may not be representative of long-term dietary patterns and may be subject to individual errors and bias in reporting. For example, a validity analysis of the USDA Automated Multiple-Pass Method used for collecting 24-h dietary recalls in WWEIA found showed obese individuals under-reported total energy intakes by about 11%, compared to just <3% for normal weight individuals [21]. NHANES is a sample of the civilian non-institutionalized U.S. population, and therefore, dietary intakes of institutionalized adults and/or active-duty personnel are not represented in these analyses. 

## Figures and Tables

**Table 1:** Nutritional biomarker data collected among various NHANES cycles.

NHANES Data Cycle	Nutrient Biomarker	N
2007–2010	Serum 4-pyridoxic acid Serum pyridoxal 5′-phosphate	15,894 15,893
2007–2012	Serum 25-dihydroxyvitamin D_2_ + D_3_	43,556
	Red blood cell (RBC) folate	16,268
2007–2014	Serum non-heme iron	35,312
	Urinary iodine	19,709
2007–2018	Serum sodium	35,352
	Serum potassium	35,344
2011–2014	Serum vitamin B_12_	9389
	Serum selenium	4386
	Serum copper	4387
	Serum zinc	4387
2017–2018	Serum retinol	6114
	Serum vitamin C	6048

**Table 2:** Pork consumption in the United States by food source.

Source	Pork Consumers		Processed Pork Consumers		Fresh Pork Consumers		Fresh Lean Pork Consumers	
Males (19+ y)	N	Mean ± SD	N	Mean ± SD	N	Mean ± SD	N	Mean ± SD
Grocery Stores	5946	80.6 ± 1.09	5208	71.0 ± 1.00	1156	94.8 ± 2.14	365	25.1 ± 0.91
Restaurants	2898	49.4 ± 1.09	2482	41.6 ± 1.08	567	70.1 ± 2.83	293	24.1 ± 0.88
Other Sources	929	64.3 ± 2.29	761	57.4 ± 2.57	201	79.8 ± 5.10	76	23.7 ± 1.56
Females (19+ y)								
Grocery Stores	5307	55.9 ± 0.82	4568	49.7 ± 0.80	1065	65.5 ± 1.76	473	26.3 ± 0.91
Restaurants	2619	35.7 ± 0.77	2229	30.7 ± 0.87	482	51.9 ± 2.14	289	22.0 ± 0.87
Other Sources	857	44.8 ± 1.76	674	39.5 ± 1.81	218	53.7 ± 3.15	116	19.4 ± 1.14
Boys (2–18 y)								
Grocery Stores	3271	58.8 ± 1.04	2975	54.9 ± 0.93	457	63.9 ± 3.52	240	26.1 ± 0.89
Restaurants	1284	26.7 ± 1.02	1150	22.3 ± 0.90	155	55.9 ± 3.52	89	25.7 ± 1.31
Schools	573	27.0 ± 1.25	559	26.2 ± 1.24	15	54.2 ± 13.3	10	22.4 ± 3.89
Other Sources	571	44.0 ± 2.05	512	40.7 ± 2.03	74	58.0 ± 6.32	40	21.2 ± 1.73
Girls (2–18 y)								
Grocery Stores	2840	49.6 ± 0.87	2571	45.9 ± 0.90	421	54.2 ± 2.22	228	24.6 ± 1.30
Restaurants	1192	24.0 ± 1.07	1059	20.8 ± 1.04	155	42.5 ± 3.03	106	22.4 ± 1.41
Schools	470	23.4 ± 1.07	460	22.7 ± 0.98	11	49.8 ± 10.5	7	22.9 ± 3.45
Other Sources	526	39.5 ± 2.04	451	36.0 ± 1.96	96	47.2 ± 4.33	62	23.5 ± 1.59

SE = standard error.

**Table 3:** Healthy Eating Index (HEI) 2015 scores in consumers vs. non-consumers of pork by gender and age.

	Pork Non-Consumer		Pork Consumer		Processed Pork Consumer		Fresh Pork Consumer		Fresh Lean Pork Consumer	
Males	N	Mean ± SE	N	Mean ± SE	N	Mean ± SE	N	Mean ± SE	N	Mean ± SE
2–3 years	155	59.9 ± 0.57	351	56.0 ± 0.49 ^‡^	338	55.9 ± 0.49 ^‡^	49	56.3 ± 0.84 ^†^	40	56.9 ± 0.94 **
4–8 years	689	55.8 ± 0.39	1985	53.9 ± 0.23 ^†^	1894	53.8 ± 0.24 ^‡^	360	55.1 ± 0.50	225	55.1 ± 0.61
9–13 years	587	54.0 ± 0.47	1844	51.5 ± 0.30 ^‡^	1744	51.4 ± 0.30 ^‡^	326	52.9 ± 0.52	145	53.7 ± 0.85
14–18 years	607	52.2 ± 0.58	1695	50.2 ± 0.27 **	1602	50.1 ± 0.27 **	307	50.3 ± 0.49 *	121	50.5 ± 0.94
19–30 years	897	56.5 ± 0.42	2242	53.3 ± 0.25 ^‡^	2050	53.1 ± 0.26 ^‡^	511	54.2 ± 0.53 ^†^	190	54.1 ± 0.80 *
31–50 years	1326	57.7 ± 0.37	3596	54.3 ± 0.21 ^‡^	3243	54.1 ± 0.21 ^‡^	1043	54.6 ± 0.30 ^‡^	385	54.5 ± 0.56 ^‡^
51–70 years	1338	59.2 ± 0.43	3686	55.1 ± 0.24 ^‡^	3324	54.9 ± 0.25 ^‡^	1061	55.0 ± 0.49 ^‡^	419	56.1 ± 0.81 **
71+ years	634	60.1 ± 0.54	1693	56.2 ± 0.28 ^‡^	1556	56.1 ± 0.28 ^‡^	421	56.2 ± 0.46 ^‡^	178	56.7 ± 0.79 ^†^
Females										
2–3 years	150	59.5 ± 0.96	330	57.9 ± 0.51	311	57.7 ± 0.53	52	59.7 ± 0.97	44	59.5 ± 1.20
4–8 years	722	58.0 ± 0.43	1766	55.3 ± 0.24 ^‡^	1674	55.2 ± 0.25 ^‡^	303	56.6 ± 0.49 *	191	57.2 ± 0.81
9–13 years	662	54.9 ± 0.41	1806	53.3 ± 0.27 **	1702	53.2 ± 0.29 **	350	53.8 ± 0.49	185	52.9 ± 0.77 *
14–18 years	732	54.2 ± 0.43	1474	52.3 ± 0.30 ^†^	1370	52.2 ± 0.32 ^†^	287	52.3 ± 0.62 *	138	53.3 ± 0.73
19–30 years	1068	59.4 ± 0.35	2198	55.6 ± 0.29 ^‡^	1992	55.3 ± 0.29 ^‡^	551	56.1 ± 0.58 ^‡^	273	56.1 ± 0.65 ^‡^
31–50 years	1807	60.9 ± 0.32	3605	56.9 ± 0.21 ^‡^	3221	56.6 ± 0.23 ^‡^	952	57.8 ± 0.34 ^‡^	460	58.2 ± 0.45 ^‡^
51–70 years	1744	61.7 ± 0.34	3394	58.0 ± 0.29 ^‡^	2992	57.7 ± 0.30 ^‡^	983	59.0 ± 0.49 ^‡^	505	58.9 ± 0.57 ^‡^
71+ years	821	61.6 ± 0.41	1558	58.6 ± 0.26 ^‡^	1398	58.3 ± 0.27 ^‡^	406	59.7 ± 0.43 ^‡^	203	60.0 ± 0.73 *

* *p* < 0.05, ** *p* < 0.01, ^†^ *p* < 0.001, ^‡^ *p* < 0.0001. SE = standard error.

## Data Availability

Publicly available datasets were analyzed in this study. The data can be found here: https://www.cdc.gov/nchs/nhanes/.

## Supplementary Materials

The following supporting information can be downloaded at: https://www.mdpi.com/article/10.3390/nu15112595/s1, Table S1: Pork consumption in the United States by DRI gender and life-stage; Table S2: Pork consumption in the United States by poverty-income-ratio; Table S3: Pork consumption in the United States by food security status; Table S4: Pork consumption in the United States by ethnicity/race; Table S5: Usual nutrient intakes from pork consumers vs. non-consumers in girls, age 2–18 years; Table S6: Usual nutrient intakes from pork consumers vs. non-consumers in boys, age 2–18 years; Table S7: Usual nutrient intakes from pork consumers vs. non-consumers in women, age 19+ years; Table S8: Usual nutrient intakes from pork consumers vs. non-consumers in men, age 19+ years; Table S9: Intake of fruits, vegetables, protein foods, meat, dairy, and added sugars in pork consumers vs. non-consumers; Table S10: Top co-consumed foods alongside pork; Table S11: Nutritional biomarker status in consumers vs. non-consumers of pork.
