# Supplementary material for: Consumption Patterns and the Nutritional Contribution of Total, Processed, Fresh, and Fresh-Lean Pork to the U.S. Diet
Source: Nutrients. 2023 Jun 1;15(11):2595. doi: 10.3390/nu15112595 (PMC10255245; doi:10.3390/nu15112595)
Supplement: Supplementary file 1 [file nutrients-15-02595-s001.zip › nutrients-2406430-supplementary.pdf]

nutrients MDPI

Table S1. Pork consumption in the United States by DRI gender and life-stage.

Gender/Age	Pork Consumers		Processed Pork Consumers		Fresh Pork Consumers		Fresh Lean Pork Consumers	
**Male**	**N**	**Mean ± SE**	**N**	**Mean ± SE**	**N**	**Mean ± SE**	**N**	**Mean ± SE**
2-3 years	635	40.3 ± 1.44	595	38.9 ± 1.54	70	34.9 ± 3.21	57	25.5 ± 1.96
4-8 years	1,537	49.6 ± 1.24	1,425	45.5 ± 1.24	237	47.8 ± 2.30	149	26.3 ± 0.98
9-13 years	1,411	55.1 ± 1.54	1,317	49.9 ± 1.60	183	65.5 ± 3.57	93	26.0 ± 1.17
14-18 years	1,311	66.9 ± 2.07	1,206	58.5 ± 1.72	197	87.3 ± 6.69	78	23.4 ± 1.42
19-30 years	1,729	81.0 ± 1.88	1,538	71.2 ± 1.62	326	93.7 ± 4.99	123	26.2 ± 1.21
31-50 years	2,755	86.8 ± 1.76	2,410	74.0 ± 1.74	647	94.2 ± 2.90	240	25.0 ± 1.03
51-70 years	2,825	77.6 ± 1.23	2,465	66.3 ± 1.17	651	85.9 ± 2.68	256	24.9 ± 0.95
70+ years	1,284	65.7 ± 1.78	1,155	57.5 ± 1.56	248	72.5 ± 3.44	104	23.4 ± 1.28
2-18 years	4,894	54.6 ± 0.93	4,543	49.4 ± 0.83	687	62.5 ± 2.59	377	25.5 ± 0.69
19+ years	8,593	79.5 ± 0.82	7,568	68.4 ± 0.79	1,872	88.3 ± 1.67	723	25.0. ± 0.55
**Female**								
2-3 years	608	37.7 ± 1.48	566	35.5 ± 1.50	88	32.2 ± 2.60	71	21.5 ± 1.78
4-8 years	1,338	46.4 ± 1.5	1,237	42.5 ± 1.52	193	48.8 ± 2.64	121	23.9 ± 1.30
9-13 years	1,354	46.7 ± 1.38	1,240	41.5 ± 1.41	207	56.8 ± 3.19	113	26.6 ± 1.62
14-18 years	1,085	49.0 ± 1.30	984	43.3 ± 1.34	177	59.3 ± 3.07	88	24.6 ± 1.38
19-30 years	1,629	55.6 ± 1.51	1,431	48.1 ± 1.55	328	66.4 ± 3.75	160	24.1 ± 1.01
31-50 years	2,661	55.8 ± 1.02	2,307	49.0 ± 1.10	579	61.2 ± 1.69	289	25.5 ± 1.20
51-70 years	2,490	54.2 ± 1.15	2,117	46.4 ± 1.03	597	61.4 ± 2.31	299	23.7 ± 1.02
70+ years	1,145	48.7 ± 1.40	1,018	42.3 ± 1.36	218	58.3 ± 3.20	114	23.9 ± 1.43
2-18 years	4,385	45.9 ± 0.73	4,027	41.4 ± 0.73	665	51.9 ± 1.63	393	24.4 ± 0.81
19+ years	7,925	54.2 ± 0.69	6,873	47.0 ± 0.69	1,722	61.9 ± 1.41	862	24.4 ± 0.68

SE = standard error.

Table S2. Pork consumption in the United States by poverty-income-ratio (PIR)

%PIR*	Pork Consumers		Processed Pork Consumers		Fresh Pork Consumers		Fresh Lean Pork Consumers	
**Adults (19+ y)**	**N**	**Mean ± SE**	**N**	**Mean ± SE**	**N**	**Mean ± SE**	**N**	**Mean ± SE**
<130	4,167	68.8 ± 1.70	3,684	59.8 ± 1.47	841	82.3 ± 5.33	356	23.8 ± 1.05
131-300	4,359	68.9 ± 1.63	3,849	59.5 ± 1.63	928	77.6 ± 3.68	390	25.2 ± 1.21
>300	4,798	69.8 ± 1.70	4,205	62.7 ± 1.72	1026	78.2 ± 2.80	485	24.7 ± 0.99
**Children (2-18 y)**								
<130	3,347	49.7 ± 1.41	3,091	44.3 ± 1.21	508	58.3 ± 2.95	276	25.5 ± 1.09
131-300	2,178	50.2 ± 2.16	2,006	45.3 ± 2.03	309	67.5 ± 7.56	181	25.1 ± 1.35
>300	1,807	56.6 ± 2.02	1,685	51.9 ± 2.09	238	68.7 ± 5.69	141	24.8 ± 1.98

SE = standard error.
*The %PIR variable is an index for the ratio of family income to poverty. The U.S. Department of Health and Human Services poverty guidelines were used as the poverty measure to calculate this index [18].

Table S3. Pork consumption in the United States by food security status.

Food Security Status	Pork Consumers		Processed Pork Consumers		Fresh Pork Consumers		Fresh Lean Pork Consumers	
**Adults (19+ y)**	**N**	**Mean ± SE**	**N**	**Mean ± SE**	**N**	**Mean ± SE**	**N**	**Mean ± SE**
Full	9,797	68.9 ± 1.27	8,595	61.2 ± 1.21	2,079	76.9 ± 2.10	933	24.2 ± 0.72
Marginal	1,634	67.6 ± 3.28	1,446	59.8 ± 3.20	341	76.3 ± 5.14	151	28.2 ± 2.16
Low	1,780	69.6 ± 2.58	1,562	58.8 ± 2.54	402	79.6 ± 4.74	174	26.6 ± 1.43
Very Low	1,071	73.3 ± 4.11	952	60.1 ± 2.60	202	100 ± 14.4	67	21.9 ± 2.05
**Children (2-18 y)**								
Full	4,455	53.9 ± 1.70	4,143	49.7 ± 1.64	583	64.9 ± 4.11	332	25.5 ± 1.11
Marginal	1,202	52.3 ± 2.52	1,103	46.2 ± 2.91	189	65.0 ± 6.72	104	25.2 ± 1.52
Low	1,422	46.5 ± 1.81	1,307	40.8 ± 1.49	237	60.0 ± 4.64	133	22.8 ± 1.43
Very Low	691	50.8 ± 3.61	632	44.1 ± 2.55	107	62.9 ± 6.21	62	26.2 ± 1.76

SE = standard error.

Table S4. Pork consumption in the United States by ethnicity/race.

	Pork Consumers		Processed Pork Consumers		Fresh Pork Consumers		Fresh Lean Pork Consumers	
**Ethnicity/Race** Both Genders; All Ages	**N**	**Mean ± SE**	**N**	**Mean ± SE**	**N**	**Mean ± SE**	**N**	**Mean ± SE**
Hispanic	6,918	55.2 ± 0.90	6,096	45.4 ± 0.78	1,446	72.6 ± 1.66	649	25.7 ± 0.59
Non-Hispanic White	9,962	65.1 ± 0.83	9,189	58.7 ± 0.80	1,447	75.7 ± 1.93	600	24.1 ± 0.57
Non-Hispanic Black	6,242	61.0 ± 0.86	5,735	54.4 ± 0.83	1,070	64.5 ± 1.99	516	22.1 ± 0.78
Non-Hispanic Asian[1]	1,419	63.1 ± 1.81	907	48.0 ± 1.87	700	65.7 ± 3.08	433	27.5 ± 1.06
Other (Includes Multi-Racial)[1]	803	63.4 ± 2.40	721	54.9 ± 2.18	142	79.7 ± 7.62	69	24.6 ± 1.42

[1]NHANES 2011-2018 data cycles only.
SE = standard error.

nutrients MDPI

Table S5: Usual nutrient intakes from pork consumers vs. non-consumers in girls, age 2-18 years.

	Pork non-consumers (N=2266)		Pork consumers (N=5376)		Processed pork consumers (N=5057)		Fresh pork consumers (N=992)		Fresh lean pork consumers (N=558)	
**Nutrient**	**Mean ± SE**	**%<EAR**	**Mean ± SE**	**%<EAR**	**Mean ± SE**	**%<EAR**	**Mean ± SE**	**%<EAR**	**Mean ± SE**	**%<EAR**
Total Calorie (Kcal)	1702 ± 11.1	--	1783 ± 6.81‡	--	1785 ± 6.98‡	--	1773 ± 12.7‡	--	1737 ± 15.8	--
Carbohydrate (g)	227 ± 1.42	1.20 ± 0.29	233 ± 0.96†	0.30 ± 0.08	233 ± 0.97†	0.30 ± 0.08	231 ± 2.00	0.50 ± 0.25	229 ± 2.25	0.50 ± 0.24
Total Fiber (g)	13.3 ± 0.11	99.7 ± 0.10	13.2 ± 0.07	99.6 ± 0.16	13.2 ± 0.07	99.6 ± 0.17	13.3 ± 0.14	99.7 ± 0.21	13.3 ± 0.16	99.7 ± 0.30
Protein (g)	60.5 ± 0.37	3.00 ± 0.57	63.4 ± 0.24‡	1.30 ± 0.23	63.4 ± 0.25‡	1.30 ± 0.23	65.0 ± 0.41‡	0.80 ± 0.33	62.7 ± 0.58**	1.50 ± 0.62
Fat (g)	64.5 ± 0.50	--	68.8 ± 0.31‡	--	69.0 ± 0.33‡	--	67.8 ± 0.51‡	--	66.3 ± 0.70*	--
Saturated Fat (g)	22.2 ± 0.20	--	23.8 ± 0.12‡	--	23.9 ± 0.12‡	--	23.5 ± 0.19‡	--	23.0 ± 0.29*	--
MUFA (g)	22.2 ± 0.19	--	23.7 ± 0.11‡	--	23.8 ± 0.12‡	--	23.5 ± 0.20‡	--	22.8 ± 0.25	--
PUFA (g)	14.6 ± 0.10	--	15.3 ± 0.07‡	--	15.3 ± 0.07‡	--	15.0 ± 0.13*	--	14.9 ± 0.16	--
Cholesterol (mg)	201 ± 1.68	--	218.5 ± 1.11‡	--	219 ± 1.18‡	--	226 ± 2.06‡	--	218 ± 2.97‡	--
Vitamin A (μg RAE)	568 ± 4.82	32.7 ± 1.50	569 ± 3.53	31.5 ± 1.06	570 ± 3.63	31.1 ± 1.08	563 ± 6.39	33.4 ± 2.33	567 ± 8.02	30.6 ± 2.52
Vitamin C (mg)	73.1 ± 0.91	23.8 ± 1.44	75.5 ± 0.78*	19.1 ± 1.03	75.4 ± 0.80*	19.0 ± 1.02	76.2 ± 1.35*	19.2 ± 2.38	77.7 ± 1.54**	13.2 ± 2.28
Vitamin D (μg)	4.80 ± 0.06	99.9 ± 0.07	5.00 ± 0.03**	100 ± 0.00	5.00 ± 0.03**	100 ± 0.00	5.00 ± 0.07*	100 ± 0.01	5.00 ± 0.11	100 ± 0.03
Vitamin E (mg)	6.50 ± 0.05	88.7 ± 1.45	6.60 ± 0.03	89.1 ± 0.57	6.60 ± 0.04	88.9 ± 0.60	6.40 ± 0.06	91.6 ± 1.16	6.40 ± 0.07	90.7 ± 1.79
Vitamin K (μg)	68.2 ± 0.64	43.3 ± 1.57	68.3 ± 0.57	39.3 ± 1.09	68.1 ± 0.58	39.6 ± 1.08	70.3 ± 0.94	38.8 ± 2.57	70.3 ± 1.10	30.6 ± 2.79
Thiamin (mg)	1.40 ± 0.01	4.30 ± 0.53	1.40 ± 0.01‡	1.90 ± 0.31	1.40 ± 0.01‡	1.80 ± 0.32	1.40 ± 0.01‡	1.10 ± 0.38	1.40 ± 0.01	1.20 ± 0.41
Riboflavin (mg)	1.70 ± 0.02	2.20 ± 0.40	1.80 ± 0.01**	0.70 ± 0.16	1.80 ± 0.01**	0.70 ± 0.16	1.80 ± 0.02	0.80 ± 0.25	1.70 ± 0.02	1.30 ± 0.46
Niacin (mg)	19.3 ± 0.13	3.50 ± 0.46	19.9 ± 0.09†	3.00 ± 0.36	19.9 ± 0.09†	3.00 ± 0.37	19.8 ± 0.15*	1.50 ± 0.47	19.1 ± 0.18	2.10 ± 0.64
Vitamin B6 (mg)	1.60 ± 0.01	4.50 ± 0.64	1.60 ± 0.01	4.20 ± 0.49	1.60 ± 0.01	4.20 ± 0.51	1.60 ± 0.01	2.70 ± 0.66	1.60 ± 0.02	4.30 ± 1.19
Folate (μg DFE)	471 ± 3.14	12.5 ± 1.00	474 ± 2.38	10.2 ± 0.79	476 ± 2.48	9.90 ± 0.79	459 ± 4.35*	13.3 ± 2.15	457 ± 5.14*	9.00 ± 1.81
Vitamin B12 (μg)	4.30 ± 0.03	2.60 ± 0.52	4.50 ± 0.02†	0.60 ± 0.15	4.50 ± 0.02‡	0.50 ± 0.16	4.40 ± 0.05	0.60 ± 0.21	4.30 ± 0.06	0.80 ± 0.35
Choline (mg)	224 ± 1.52	91.8 ± 1.08	237 ± 0.99‡	88.5 ± 0.61	237 ± 1.04‡	88.2 ± 0.65	246 ± 1.98‡	89.8 ± 1.11	237 ± 2.66†	89.5 ± 1.68
Calcium (mg)	900 ± 7.58	80.9 ± 1.43	934 ± 4.94†	80.6 ± 0.74	937 ± 5.01‡	80.3 ± 0.78	906 ± 9.74	84.2 ± 1.59	907 ± 12.8	80.1 ± 2.58
Potassium (mg)	1954 ± 11.4	81.5 ± 1.37	2023 ± 8.56‡	77.0 ± 0.98	2024 ± 8.75‡	77.1 ± 1.01	2056 ± 15.3‡	74.4 ± 2.09	2010 ± 21.3*	77.9 ± 2.22

Sodium (mg)	2638 ± 18.1	0.00 ± 0.02	2867 ± 10.9‡	0.00 ± 0.00	2873 ± 11.3‡	0.00 ± 0.00	2856 ± 22.1‡	0.00 ± 0.00	2818 ± 31.9‡	0.00 ± 0.00
Phosphorus (mg)	1108 ± 7.96	0.00 ± 0.00	1163 ± 4.66‡	0.00 ± 0.00	1165 ± 4.73‡	0.00 ± 0.00	1168 ± 8.99‡	0.00 ± 0.00	1142 ± 12.6*	0.00 ± 0.00
Iron (mg)	12.6 ± 0.08	35.7 ± 1.44	12.9 ± 0.06*	29.1 ± 0.94	12.9 ± 0.06**	28.9 ± 0.98	12.5 ± 0.11	31.4 ± 2.29	12.5 ± 0.14	26.0 ± 3.05
Magnesium (mg)	217 ± 1.40	57.2 ± 1.81	219 ± 1.13	53.1 ± 1.07	219 ± 1.15	52.7 ± 1.11	220 ± 1.81	57.3 ± 2.25	215 ± 2.35	48.8 ± 2.94
Zinc (mg)	8.90 ± 0.06	29.4 ± 1.46	9.20 ± 0.04‡	20.8 ± 0.89	9.30 ± 0.04‡	20.3 ± 0.90	9.30 ± 0.08‡	22.1 ± 2.04	9.10 ± 0.10	21.8 ± 2.61
Copper (mg)	0.90 ± 0.01	19.9 ± 1.50	0.90 ± 0.00	15.2 ± 0.68	0.90 ± 0.00	14.8 ± 0.67	0.90 ± 0.01	15.1 ± 1.67	0.90 ± 0.01	13.6 ± 1.70
Selenium (mg)	84.4 ± 0.51	0.60 ± 0.19	89.0 ± 0.34‡	0.40 ± 0.15	89.0 ± 0.35‡	0.40 ± 0.16	91.6 ± 0.67‡	0.10 ± 0.10	88.7 ± 0.93†	0.20 ± 0.18

*$p<0.05$, **$p<0.01$, †$p<0.001$, ‡$p<0.0001$
SE = standard error.

Table S6: Usual nutrient intakes from pork consumers vs. non-consumers in boys, age 2-18 years.

	Pork non-consumers (N=2038)		Pork consumers (N=5875)		Processed pork consumers (N=5578)		Fresh pork consumers (N=1042)		Fresh lean pork consumers (N=531)	
**Nutrient**	**Mean ± SE**	**%<EAR**	**Mean ± SE**	**%<EAR**	**Mean ± SE**	**%<EAR**	**Mean ± SE**	**%<EAR**	**Mean ± SE**	**%<EAR**
Total Calorie (Kcal)	2061 ± 15.4	--	2158 ± 8.38‡	--	2161 ± 8.76‡	--	2152 ± 18.0†	--	2067 ± 20.0	--
Carbohydrate (g)	270 ± 2.05	0.30 ± 0.18	276 ± 1.17**	0.00 ± 0.01	277 ± 1.22**	0.00 ± 0.02	274 ± 2.63	0.00 ± 0.01	266 ± 2.58	0.00 ± 0.02
Total Fiber (g)	15.0 ± 0.17	99.4 ± 0.24	15.0 ± 0.08	99.7 ± 0.10	15.0 ± 0.08	99.7 ± 0.11	15.2 ± 0.17	99.9 ± 0.07	14.8 ± 0.19	99.8 ± 0.13
Protein (g)	75.4 ± 0.55	0.60 ± 0.23	79.3 ± 0.30‡	0.30 ± 0.10	79.3 ± 0.30‡	0.30 ± 0.10	81.7 ± 0.68‡	0.10 ± 0.05	77.1 ± 0.80	0.1- ± 0.11
Fat (g)	77.2 ± 0.66	--	82.7 ± 0.36‡	--	83.0 ± 0.38‡	--	81.6 ± 0.73‡	--	79.0 ± 0.95	--
Saturated Fat (g)	26.6 ± 0.23	--	28.9 ± 0.14‡	--	29.0 ± 0.15‡	--	28.3 ± 0.28‡	--	27.5 ± 0.43	--
MUFA (g)	26.9 ± 0.24	--	28.9 ± 0.13‡	--	28.9 ± 0.13‡	--	28.6 ± 0.26‡	--	27.4 ± 0.31	--
PUFA (g)	17.0 ± 0.15	--	17.7 ± 0.08‡	--	17.8 ± 0.08‡	--	17.6 ± 0.15*	--	17.2 ± 0.19	--
Cholesterol (mg)	253 ± 2.09	--	273 ± 1.39‡	--	273 ± 1.45‡	--	281 ± 2.82‡	--	271 ± 3.98†	--
Vitamin A (µg RAE)	634 ± 7.37	49.7 ± 1.90	644 ± 4.04	47.4 ± 1.13	645 ± 4.19	47.1 ± 1.15	630 ± 6.75	51.9 ± 2.03	627 ± 11.8	53.2 ± 3.05
Vitamin C (mg)	78.7 ± 1.31	22.0 ± 1.85	80.2 ± 0.81	20.8 ± 0.99	79.9 ± 0.79	20.9 ± 1.00	82.9 ± 1.85*	19.1 ± 2.11	81.7 ± 1.96	14.9 ± 2.85
Vitamin D (µg)	5.90 ± 0.08	100 ± 0.00	6.10 ± 0.04	100 ± 0.03	6.10 ± 0.05*	100 ± 0.02	6.10 ± 0.08	99.9 ± 0.12	6.10 ± 0.15	99.7 ± 0.26
Vitamin E (mg)	7.60 ± 0.13	78.3 ± 1.46	7.40 ± 0.04	79.5 ± 0.86	7.50 ± 0.04	79.5 ± 0.93	7.40 ± 0.07	81.2 ± 1.67	7.20 ± 0.09*	79.2 ± 2.22
Vitamin K (µg)	70.2 ± 0.71	34.2 ± 1.98	71.9 ± 0.50*	30.5 ± 1.09	71.6 ± 0.51	30.9 ± 1.13	75.3 ± 1.07‡	28.1 ± 2.18	74.1 ± 1.10**	26.4 ± 2.61
Thiamin (mg)	1.70 ± 0.01	1.60 ± 0.45	1.70 ± 0.01‡	1.30 ± 0.23	1.70 ± 0.01‡	1.30 ± 0.24	1.80 ± 0.01‡	1.00 ± 0.48	1.70 ± 0.02	0.70 ± 0.52
Riboflavin (mg)	2.10 ± 0.03	1.00 ± 0.28	2.20 ± 0.01*	0.80 ± 0.15	2.20 ± 0.01*	0.80 ± 0.15	2.20 ± 0.03	0.80 ± 0.33	2.10 ± 0.04	1.20 ± 0.63
Niacin (mg)	24.7 ± 0.25	0.30 ± 0.11	25.1 ± 0.11	0.40 ± 0.10	25.1 ± 0.11	0.40 ± 0.11	25.5 ± 0.24*	0.10 ± 0.05	23.9 ± 0.27	0.10 ± 0.12
Vitamin B6 (mg)	2.00 ± 0.02	1.70 ± 0.54	2.00 ± 0.01	1.00 ± 0.20	2.00 ± 0.01	1.00 ± 0.21	2.10 ± 0.02	0.80 ± 0.43	2.00 ± 0.03	0.20 ± 0.14
Folate (µg DFE)	558 ± 6.08	3.70 ± 0.88	569 ± 2.80	2.20 ± 0.28	570 ± 2.91*	2.10 ± 0.29	564 ± 5.65	1.90 ± 0.44	548 ± 7.69	1.60 ± 0.63
Vitamin B12 (µg)	5.60 ± 0.06	0.10 ± 0.06	5.80 ± 0.03*	0.10 ± 0.09	5.80 ± 0.03**	0.10 ± 0.10	5.70 ± 0.06	0.00 ± 0.00	5.50 ± 0.10	0.00 ± 0.00
Choline (mg)	281 ± 2.43	73.3 ± 1.47	297 ± 1.18‡	69.1 ± 1.02	297 ± 1.22‡	68.9 ± 1.05	307 ± 2.93‡	69.5 ± 2.17	294 ± 3.73**	62.2 ± 3.19
Calcium (mg)	1048 ± 11.3	64.5 ± 1.74	1110 ± 5.75‡	59.0 ± 1.09	1112 ± 6.02‡	58.6 ± 1.16	1074 ± 12.1	65.7 ± 2.29	1056 ± 18.1	62.7 ± 3.31
Potassium (mg)	2315 ± 22.6	70.3 ± 1.86	2400 ± 10.8†	63.2 ± 1.01	2397 ± 10.9†	63.3 ± 1.00	2468 ± 26.9‡	59.4 ± 2.27	2352 ± 27.7	61.9 ± 3.10
Sodium (mg)	3197 ± 25.2	0.00 ± 0.00	3507 ± 14.6‡	0.00 ± 0.00	3517 ± 15.1‡	0.00 ± 0.00	3526 ± 34.3‡	0.00 ± 0.00	3361 ± 36.3**	0.00 ± 0.00

Phosphorus (mg)	1347 ± 11.8	0.00 ± 0.00	1417 ± 5.70‡	0.00 ± 0.00	1419 ± 5.93‡	0.00 ± 0.00	1422 ± 12.4‡	0.00 ± 0.00	1367 ± 15.5	0.00 ± 0.00
Iron (mg)	15.3 ± 0.16	3.60 ± 0.79	15.6 ± 0.06	1.70 ± 0.23	15.7 ± 0.06*	1.60 ± 0.23	15.5 ± 0.12	1.90 ± 0.50	15.0 ± 0.18	2.10 ± 0.73
Magnesium (mg)	254 ± 2.82	44.7 ± 2.12	256 ± 1.18	42.9 ± 1.40	256 ± 1.21	42.9 ± 1.42	261 ± 2.61	43.8 ± 2.48	249 ± 2.94	35.3 ± 3.46
Zinc (mg)	11.2 ± 0.11	15.7 ± 1.62	11.6 ± 0.05‡	10.2 ± 0.79	11.7 ± 0.05‡	10.2 ± 0.82	11.8 ± 0.10‡	8.80 ± 1.20	11.2 ± 0.15	8.90 ± 1.70
Copper (mg)	1.00 ± 0.01	11.2 ± 1.60	1.10 ± 0.01**	5.40 ± 0.42	1.10 ± 0.01**	5.40 ± 0.43	1.10 ± 0.01**	3.60 ± 0.79	1.00 ± 0.01	3.20 ± 1.03
Selenium (mg)	104 ± 0.80	0.00 ± 0.03	111 ± 0.42‡	0.00 ± 0.00	111 ± 0.43‡	0.00 ± 0.00	115 ± 0.93‡	0.00 ± 0.00	109 ± 1.12*	0.00 ± 0.00

*p<0.05, **p<0.01, †p<0.001, ‡p<0.0001
SE = standard error.

Table S7: Usual nutrient intakes from pork consumers vs. non-consumers in women, age 19+ years.

	Pork non-consumers (N=5440)		Pork consumers (N=10755)		Processed pork consumers (N=9603)		Fresh pork consumers (N=2892)		Fresh lean pork consumers (N=1441)	
**Nutrient**	**Mean ± SE**	**%<EAR**	**Mean ± SE**	**%<EAR**	**Mean ± SE**	**%<EAR**	**Mean ± SE**	**%<EAR**	**Mean ± SE**	**%<EAR**
Total Calorie (Kcal)	1738 ± 8.03	--	1846 ± 4.82‡	--	1852 ± 5.14‡	--	1837 ± 7.36‡	--	1804 ± 12.5‡	--
Carbohydrate (g)	216 ± 1.16	2.60 ± 0.35	223 ± 0.69‡	1.10 ± 0.15	224 ± 0.72‡	1.10 ± 0.17	223 ± 1.12‡	0.90 ± 0.19	222 ± 1.79**	1.40 ± 0.40
Total Fiber (g)	15.6 ± 0.13	92.2 ± 0.62	14.9 ± 0.07‡	95.9 ± 0.31	14.9 ± 0.07‡	96.1 ± 0.31	15.3 ± 0.12*	95.5 ± 0.52	15.1 ± 0.17**	95.9 ± 0.55
Protein (g)	66.8 ± 0.29	3.80 ± 0.46	70.4 ± 0.18‡	1.60 ± 0.13	70.4 ± 0.19‡	1.50 ± 0.14	71.7 ± 0.27‡	1.10 ± 0.19	69.5 ± 0.51‡	2.00 ± 0.38
Fat (g)	66.7 ± 0.35	--	72.5 ± 0.24‡	--	73.0 ± 0.24‡	--	71.0 ± 0.36‡	--	69.4 ± 0.56‡	--
Saturated Fat (g)	21.4 ± 0.12	--	23.7 ± 0.09‡	--	23.9 ± 0.09‡	--	22.9 ± 0.13‡	--	22.3 ± 0.19‡	--
MUFA (g)	23.4 ± 0.13	--	25.6 ± 0.09‡	--	25.7 ± 0.09‡	--	25.2 ± 0.13‡	--	24.6 ± 0.20‡	--
PUFA (g)	15.9 ± 0.09	--	16.8 ± 0.06‡	--	16.9 ± 0.06‡	--	16.5 ± 0.10‡	--	16.4 ± 0.15*	--
Cholesterol (mg)	234 ± 1.23	--	259 ± 0.99‡	--	260 ± 1.03‡	--	262 ± 1.49‡	--	257 ± 2.28‡	--
Vitamin A (µg RAE)	591 ± 5.15	31.8 ± 1.17	580 ± 3.09*	32.9 ± 0.96	581 ± 3.20*	32.8 ± 0.98	573 ± 5.08**	34.4 ± 1.57	566 ± 6.76**	35.2 ± 2.05
Vitamin C (mg)	80.1 ± 0.75	48.7 ± 1.20	76.0 ± 0.59‡	54.2 ± 0.86	75.4 ± 0.59‡	55.2 ± 0.88	79.7 ± 1.05	48.0 ± 1.47	79.7 ± 1.36	47.1 ± 2.01
Vitamin D (µg)	4.10 ± 0.03	100 ± 0.01	4.10 ± 0.02	100 ± 0.00	4.10 ± 0.02	100 ± 0.00	4.20 ± 0.03*	100 ± 0.00	4.00 ± 0.05	100 ± 0.00
Vitamin E (mg)	7.70 ± 0.06	99.0 ± 0.23	7.60 ± 0.04	99.3 ± 0.13	7.60 ± 0.04	99.3 ± 0.14	7.40 ± 0.05**	99.3 ± 0.32	7.20 ± 0.07‡	100 ± 0.03
Vitamin K (µg)	103 ± 0.86	43.4 ± 1.18	98.7 ± 0.63†	46.3 ± 0.90	98.1 ± 0.66†	47.2 ± 0.92	102 ± 1.08	40.0 ± 1.41	104 ± 1.54	36.9 ± 2.10
Thiamin (mg)	1.30 ± 0.01	18.0 ± 0.84	1.40 ± 0.00‡	9.40 ± 0.48	1.40 ± 0.00‡	9.40 ± 0.48	1.50 ± 0.01‡	6.60 ± 0.73	1.40 ± 0.01‡	10.5 ± 1.32
Riboflavin (mg)	1.80 ± 0.01	4.9.0 ± 0.44	1.90 ± 0.01‡	2.30 ± 0.18	1.90 ± 0.01‡	2.20 ± 0.18	1.80 ± 0.01**	2.70 ± 0.40	1.80 ± 0.02	3.90 ± 0.71
Niacin (mg)	20.8 ± 0.10	0.00 ± 0.00	21.5 ± 0.05‡	0.00 ± 0.00	21.6 ± 0.06‡	0.00 ± 0.00	21.7 ± 0.10‡	0.00 ± 0.00	21.0 ± 0.17	0.00 ± 0.00
Vitamin B6 (mg)	1.70 ± 0.01	19.1 ± 1.04	1.80 ± 0.01	15.5 ± 0.55	1.80 ± 0.01	15.6 ± 0.56	1.80 ± 0.01**	12.7 ± 0.90	1.70 ± 0.02	17.1 ± 1.61
Folate (µg DFE)	462 ± 3.00	30.0 ± 0.98	462 ± 1.73	28.8 ± 0.76	462 ± 1.76	28.8 ± 0.77	458 ± 3.29	28.0 ± 1.14	454 ± 3.91	29.6 ± 1.73
Vitamin B12 (µg)	4.10 ± 0.03	5.40 ± 0.47	4.30 ± 0.02†	1.70 ± 0.20	4.30 ± 0.02‡	1.60 ± 0.21	4.20 ± 0.03	2.00 ± 0.29	4.10 ± 0.04	2.40 ± 0.49
Choline (mg)	271 ± 1.21	99.1 ± 0.23	286 ± 1.01‡	98.6 ± 0.17	286 ± 1.06‡	98.6 ± 0.17	293 ± 1.46‡	98.0 ± 0.46	286 ± 2.13‡	98.4 ± 0.61
Calcium (mg)	831 ± 5.25	86.4 ± 0.76	859 ± 3.54‡	83.2 ± 0.57	864 ± 3.74‡	82.7 ± 0.61	829 ± 4.77	85.8 ± 1.07	808 ± 7.48**	87.9 ± 1.49
Potassium (mg)	2312 ± 12.0	74.3 ± 0.94	2334 ± 9.12	73.4 ± 0.77	2331 ± 9.52	73.6 ± 0.83	2372 ± 14.2†	71.3 ± 1.28	2304 ± 19.8	76.2 ± 1.61
Sodium (mg)	2790 ± 12.7	0.80 ± 0.18	3077 ± 8.13‡	0.10 ± 0.04	3088 ± 8.47‡	0.10 ± 0.03	3079 ± 15.3‡	0.30 ± 0.12	3046 ± 24.9‡	0.60 ± 0.25

Phosphorus (mg)	1141 ± 5.93	0.00 ± 0.00	1198 ± 3.74‡	0.00 ± 0.00	1200 ± 4.04‡	0.00 ± 0.00	1194 ± 4.57‡	0.00 ± 0.00	1162 ± 8.63*	0.00 ± 0.00
Iron (mg)	12.7 ± 0.08	55.4 ± 1.07	13.0 ± 0.04**	55.3 ± 0.82	13.0 ± 0.04**	55.6 ± 0.86	12.9 ± 0.07	55.4 ± 1.40	12.70 ± 0.10	54.8 ± 1.86
Magnesium (mg)	266 ± 1.77	80.7 ± 0.97	260 ± 1.12†	84.6 ± 0.64	260 ± 1.19†	84.8 ± 0.65	263 ± 1.52	84.4 ± 1.14	258 ± 2.66**	85.0 ± 1.52
Zinc (mg)	9.40 ± 0.05	45.1 ± 1.21	9.70 ± 0.03‡	36.4 ± 0.75	9.70 ± 0.03‡	36.5 ± 0.80	9.80 ± 0.04‡	33.0 ± 1.24	9.50 ± 0.07	39.3 ± 1.98
Copper (mg)	1.10 ± 0.01	21.1 ± 0.91	1.10 ± 0.00	20.1 ± 0.72	1.10 ± 0.00	20.4 ± 0.75	1.10 ± 0.01	18.2 ± 1.09	1.10 ± 0.01	20.1 ± 1.65
Selenium (mg)	92.7 ± 0.43	1.30 ± 0.21	98.3 ± 0.24‡	0.30 ± 0.09	98.3 ± 0.25‡	0.30 ± 0.10	101 ± 0.42‡	0.30 ± 0.12	97.6 ± 0.74‡	0.50 ± 0.25

*p<0.05, **p<0.01, †p<0.001, ‡p<0.0001

SE = standard error.

Table S8: Usual nutrient intakes from pork consumers vs. non-consumers in men, age 19+ years.

	Pork non-consumers (N=4195)		Pork consumers (N=11217)		Processed pork consumers (N=10173)		Fresh pork consumers (N=3036)		Fresh lean pork consumers (N=1172)	
**Nutrient**	**Mean ± SE**	**%<EAR**	**Mean ± SE**	**%<EAR**	**Mean ± SE**	**%<EAR**	**Mean ± SE**	**%<EAR**	**Mean ± SE**	**%<EAR**
Total Calorie (Kcal)	2240 ± 13.4	--	2409 ± 7.58‡	--	2422 ± 7.88‡	--	2409 ± 13.6‡	--	2371 ± 21.8‡	--
Carbohydrate (g)	271 ± 1.90	1.00 ± 0.22	282 ± 1.00‡	0.40 ± 0.10	283 ± 1.01‡	0.40 ± 0.11	282 ± 1.85†	0.20 ± 0.11	283 ± 2.93**	0.20 ± 0.14
Total Fiber (g)	18.1 ± 0.19	97.9 ± 0.38	17.8 ± 0.09	99.0 ± 0.14	17.7 ± 0.09*	99.1 ± 0.14	18.0 ± 0.18	98.7 ± 0.31	18.3 ± 0.30	98.0 ± 0.71
Protein (g)	87.6 ± 0.51	2.80 ± 0.44	93,0 ± 0.26‡	0.80 ± 0.11	93.2 ± 0.27‡	0.70 ± 0.10	94.8 ± 0.48‡	0.80 ± 0.36	92.3 ± 0.82‡	1.80 ± 0.95
Fat (g)	83.7 ± 0.54	--	93.2 ± 0.33‡	--	94.0 ± 0.35‡	--	92.0 ± 0.60‡	--	89.9 ± 0.98‡	--
Saturated Fat (g)	27.0 ± 0.18	--	30.6 ± 0.12‡	--	30.9 ± 0.13‡	--	29.9 ± 0.24‡	--	29.0 ± 0.37‡	--
MUFA (g)	29.8 ± 0.20	--	33.3 ± 0.12‡	--	33.5 ± 0.13‡	--	33.0 ± 0.21‡	--	32.1 ± 0.33‡	--
PUFA (g)	19.1 ± 0.13	--	20.6 ± 0.07‡	--	20.7 ± 0.07‡	--	20.4 ± 0.13‡	--	20.3 ± 0.22‡	--
Cholesterol (mg)	303 ± 2.09	--	340 ± 1.31‡	--	342 ± 1.36‡	--	345 ± 2.21‡	--	339 ± 3.75‡	--
Vitamin A (μg RAE)	635 ± 6.46	52.1 ± 1.46	651 ± 3.49*	49.0 ± 0.88	656 ± 3.59**	47.7 ± 0.88	628 ± 7.34	55.5 ± 1.71	630 ± 10.7	53.6 ± 2.42
Vitamin C (mg)	84.4 ± 1.03	62.2 ± 1.21	82.0 ± 0.61*	65.2 ± 0.80	81.7 ± 0.63*	65.5 ± 0.85	83.7 ± 1.11	63.3 ± 1.43	84.9 ± 1.87	63.0 ± 2.37
Vitamin D (μg)	4.80 ± 0.06	99.8 ± 0.10	5.00 ± 0.03†	100 ± 0.02	5.10 ± 0.03‡	100 ± 0.02	5.00 ± 0.06*	100 ± 0.00	4.80 ± 0.08	100 ± 0.00
Vitamin E (mg)	8.80 ± 0.09	97.1 ± 0.45	8.90 ± 0.04	97.7 ± 0.23	9.00 ± 0.04	97.7 ± 0.24	8.80 ± 0.07	98 ± 0.40	8.70 ± 0.11	98.9 ± 0.52
Vitamin K (μg)	104 ± 1.31	75.7 ± 1.37	104 ± 0.54	74.6 ± 0.68	104 ± 0.58	74.8 ± 0.70	106 ± 0.90	73.3 ± 1.42	110 ± 1.57**	69.5 ± 2.29
Thiamin (mg)	1.70 ± 0.01	7.30 ± 0.60	1.80 ± 0.01‡	1.90 ± 0.19	1.80 ± 0.01‡	1.80 ± 0.19	1.90 ± 0.01‡	1.30 ± 0.25	1.80 ± 0.02‡	2.30 ± 0.55
Riboflavin (mg)	2.20 ± 0.02	4.40 ± 0.46	2.40 ± 0.01‡	1.20 ± 0.13	2.40 ± 0.01‡	1.10 ± 0.13	2.30 ± 0.02†	1.30 ± 0.25	2.30 ± 0.03	2.10 ± 0.54
Niacin (mg)	27.9 ± 0.19	1.40 ± 0.31	29.4 ± 0.10‡	0.40 ± 0.07	29.5 ± 0.11‡	0.40 ± 0.07	29.5 ± 0.18‡	0.20 ± 0.09	28.6 ± 0.28*	0.40 ± 0.14
Vitamin B6 (mg)	2.30 ± 0.02	6.50 ± 0.64	2.30 ± 0.01*	4.60 ± 0.28	2.30 ± 0.01*	4.60 ± 0.31	2.40 ± 0.02**	3.80 ± 0.62	2.30 ± 0.03	5.30 ± 1.16
Folate (μg DFE)	569 ± 4.46	9.90 ± 0.76	582 ± 2.15**	5.80 ± 0.35	585 ± 2.22**	5.30 ± 0.37	571 ± 4.12	8.00 ± 0.87	574 ± 6.84	8.20 ± 1.46
Vitamin B12 (μg)	5.50 ± 0.05	1.60 ± 0.23	5.70 ± 0.03‡	0.20 ± 0.06	5.80 ± 0.03‡	0.20 ± 0.06	5.60 ± 0.06	0.20 ± 0.08	5.60 ± 0.09	0.20 ± 0.10
Choline (mg)	351 ± 1.96	98.8 ± 0.27	380 ± 1.17‡	97.1 ± 0.23	381 ± 1.23‡	97.0 ± 0.25	390 ± 2.17‡	95.8 ± 0.61	381 ± 3.43‡	96.5 ± 0.96
Calcium (mg)	993 ± 8.20	58.2 ± 1.62	1045 ± 4.61‡	49.9 ± 0.84	1056 ± 4.59‡	48.0 ± 0.86	996 ± 10.6	58.8 ± 1.74	986 ± 16.2	61.0 ± 2.45
Potassium (mg)	2794 ± 19.9	84.0 ± 1.07	2901 ± 10.4‡	81.0 ± 0.68	2906 ± 11.0‡	80.7 ± 0.71	2938 ± 21.3‡	80.0 ± 1.36	2863 ± 29.2	80.8 ± 1.74
Sodium (mg)	3578 ± 19.7	0.30 ± 0.09	4008 ± 11.9‡	0.00 ± 0.01	4036 ± 12.4‡	0.00 ± 0.01	3996 ± 19.3‡	0.00 ± 0.00	4002 ± 35.3‡	0.00 ± 0.00

Phosphorus (mg)	1452 ± 9.51	0.00 ± 0.00	1548 ± 4.92‡	0.00 ± 0.00	1557 ± 5.28‡	0.00 ± 0.00	1544 ± 10.0‡	0.00 ± 0.00	1506 ± 16.5**	0.00 ± 0.00
Iron (mg)	16.0 ± 0.11	0.90 ± 0.23	16.6 ± 0.05‡	0.20 ± 0.05	16.7 ± 0.05‡	0.20 ± 0.05	16.4 ± 0.10*	0.20 ± 0.10	16.4 ± 0.18	0.40 ± 0.24
Magnesium (mg)	320 ± 2.77	88.5 ± 0.95	324 ± 1.31	89.3 ± 0.47	323 ± 1.36	89.3 ± 0.50	328 ± 2.65	88.5 ± 0.89	322 ± 4.32	89.1 ± 1.39
Zinc (mg)	12.2 ± 0.08	34.1 ± 1.35	12.9 ± 0.04‡	23.1 ± 0.67	12.9 ± 0.04‡	22.4 ± 0.67	13.0 ± 0.08‡	21.5 ± 1.21	12.9 ± 0.15†	25.6 ± 2.05
Copper (mg)	1.30 ± 0.01	9.60 ± 0.76	1.30 ± 0.01**	5.30 ± 0.29	1.30 ± 0.01**	5.00 ± 0.32	1.40 ± 0.01**	4.80 ± 0.62	1.40 ± 0.02*	6.20 ± 1.13
Selenium (mg)	120 ± 0.76	0.40 ± 0.11	130 ± 0.34‡	0.10 ± 0.04	130 ± 0.36‡	0.10 ± 0.04	133 ± 0.80‡	0.00 ± 0.00	131 ± 1.67‡	0.00 ± 0.00

*p<0.05, **p<0.01, †p<0.001, ‡p<0.0001
SE = standard error.

Table S9. Intake of fruits, vegetables, grains, protein foods, meat, dairy, and added sugars in pork consumers and non-consumers.

	Non-consumers		Pork Consumers		Processed Pork Consumers		Fresh Pork Consumers		Fresh Lean Pork Consumers	
**Food Groups**	N	**Mean ± SE**	N	**Mean ± SE**	N	**Mean ± SE**	N	**Mean ± SE**	N	**Mean ± SE**
Fruit (cup eq.)	11,195	1.25 ± 0.02	27,528	1.15 ± 0.01‡	25,354	1.14 ± 0.01‡	6,544	1.15 ± 0.02‡	3,182	1.15 ± 0.03‡
Vegetables (cup eq.)	12,236	1.44 ± 0.02	31,249	1.39 ± 0.01‡	28,797	1.37 ± 0.01‡	7,451	1.52 ± 0.02‡	3,553	1.44 ± 0.03
Dark green vegetables (cup eq.)	4,353	0.41 ± 0.01	9,701	0.36 ± 0.01‡	8,611	0.36 ± 0.01‡	2,874	0.35 ± 0.01‡	1,479	0.33 ± 0.01‡
Whole grains (ounce eq.)	9,143	1.31 ± 0.03	22,680	1.15 ± 0.02‡	21,070	1.15 ± 0.02‡	5,043	1.09 ± 0.03‡	2,442	1.03 ± 0.03‡
Refined grains (ounce eq.)	12,471	4.88 ± 0.04	31,451	5.80 ± 0.04‡	28,984	5.82 ± 0.03‡	7,482	5.87 ± 0.06‡	3,569	5.79 ± 0.09‡
Total protein foods (ounce eq.)	11,689	4.77 ± 0.07	31,196	5.98 ± 0.04‡	28,798	5.96 ± 0.04‡	7,378	6.69 ± 0.07‡	3,513	5.87 ± 0.12‡
Meat (ounce eq.)	6,714	2.09 ± 0.04‡	22,662	2.13 ± 0.02‡	20,189	2.06 ± 0.02‡	7,494	2.65 ± 0.04‡	3,571	1.85 ± 0.05‡
Total dairy (cup eq.)	12,984	1.52 ± 0.03‡	31,500	1.73 ± 0.02‡	29,027	1.77 ± 0.02‡	7,494	1.50 ± 0.03‡	3,571	1.50 ± 0.04‡
Added sugars (g)	12,293	14.22 ± 0.19‡	31,474	17.2 ± 0.16‡	29,010	17.4 ± 0.17‡	7,482	17.0 ± 0.28‡	3,567	16.0 ± 0.37‡

‡$p<0.0001$ compared to non-consumers.
SE = standard error.

Table S10. Top co-consumed foods alongside pork.

Pork Consumers		Processed Pork Consumers		Fresh Pork Consumers		Fresh Lean Pork Consumers	
1.	Lettuce, raw	1.	Lettuce, raw	1.	Lettuce, raw	1.	Lettuce, raw
2.	Tomatoes, raw	2.	Tomatoes, raw	2.	Tomatoes, raw	2.	Tomatoes, raw
3.	Ketchup	3.	Ketchup	3.	Banana, raw	3.	Salsa, red
4.	Mayonnaise, regular	4.	Mayonnaise, regular	4.	Salsa, red	4.	Banana, raw
5.	Mustard	5.	Mustard	5.	Apple, raw	5.	Soy sauce
6.	Bread, white	6.	Bread, white	6.	Ketchup	6.	Apple, raw
7.	Banana, raw	7.	Banana, raw	7.	Rice, white, cooked	7.	Ketchup
8.	Apple, raw	8.	Roll, white, soft	8.	Mayonnaise, regular	8.	Rice, white, cooked
9.	Roll, white, soft	9.	Apple, raw	9.	Bread, white	9.	Mustard
10.	Cheese, American	10.	Cheese, American	10.	Roll, white, soft	10.	Mayonnaise, regular

Table S11. Nutritional biomarker status in consumers and non-consumers of pork.

	Non-Consumers		Pork Consumers		Processed Pork Consumers		Fresh Pork Consumers		Fresh Lean Pork	
**Nutrient**	**N**	**Mean ± SD**	**N**	**Mean ± SD**	**N**	**Mean ± SD**	**N**	**Mean ± SD**	**N**	**Mean ± SD**
Retinol (umol/L)	2,933	1.72 ± 0.01	3,181	1.74 ± 0.02	2,767	1.74 ± 0.02	682	1.7 ± 0.04	327	1.68 ± 0.07
Vitamin B12 (pmol/L)	4,334	398 ± 4.84	5,055	379 ± 2.76†	4,399	377 ± 2.66‡	1,157	391 ± 7.39	530	403 ± 12.2
Pyridoxal 5'-phosphate (nmol/L)	7,526	52.3 ± 0.89	8,367	51.6 ± 1.22	7,625	51.4 ± 1.16	1,356	51.9 ± 1.93	598	50.8 ± 2.78
4-pyridoxic acid (nmol/L)	7,530	29.9 ± 0.55	8,364	29.1 ± 0.59	7,623	29.0 ± 0.54*	1,355	29.2 ± 1.33	598	28.3 ± 2.08
RBC folate (nmol/L)	7,725	1080 ± 13.2	8,543	1079 ± 14.5	7,793	1083 ± 13.93	1,380	1052 ± 27.3	611	1013 ± 34.9*
Vitamin C (umol/L)	2,903	44.7 ± 1.58	3,145	43.0 ± 1.62	2,737	42.8 ± 1.6	670	44.8 ± 2.47	321	46.0 ± 2.54
25OHD2+25OHD3 (nmol/L)	20,358	65.1 ± 0.61	23,198	64.1 ± 0.65**	20,643	64.3 ± 0.65	4,533	61.8 ± 0.81‡	2,128	61.9 ± 0.94**
alpha-tocopherol (umol/L)	2,866	1144 ± 8.51	3,115	1108 ± 13.7†	2,711	1109 ± 14.6**	662	1109 ± 15.5	313	1082 ± 20.0*
Serum Copper (umol/L)	1,974	116 ± 1.05	2,413	113 ± 0.99†	2,104	113 ± 1.02†	512	111 ± 2.00	252	112 ± 2.07
Serum Selenium (umol/L)	1,974	127 ± 1.05	2,412	128 ± 0.78	2,103	128 ± 0.85	512	129 ± 1.33	252	127 ± 1.38
Serum Zinc (umol/L)	1,974	81.1 ± 0.69	2,413	81.4 ± 0.65	2,104	81.5 ± 0.67	512	80.8 ± 1.01	252	80.9 ± 1.32
Iodine, urine (ug/L)	9,135	138 ± 2.51	10,574	143 ± 2.69	9,432	144 ± 2.71*	1,981	133.6 ± 5.03	892	135 ± 7.19
Potassium (mmol/L)	16,713	3.98 ± 0.01	18,631	3.99 ± 0.01	16,420	3.99 ± 0.01	3,844	3.98 ± 0.01	1,684	3.98 ± 0.01
Sodium (mmol/L)	16,715	139 ± 0.09	18,637	139 ± 0.09	16,425	139 ± 0.09	3,845	139 ± 0.1	1,685	139 ± 0.14
Iron, refrigerated (ug/dL)	16,694	78.7 ± 0.46	18618	79.8 ± 0.42*	16,408	79.8 ± 0.44	3,842	79.8 ± 0.74	1,684	79.5 ± 1.14

*p<0.05, **p<0.01, †p<0.001, ‡p<0.0001
SE = standard error.
